# Effect of Self-Care Activities and Behaviors on Glycemic Control in Patients With Diabetes at a Tertiary Care Hospital in Perambalur, South India: A Cross-Sectional Study

**DOI:** 10.7759/cureus.52188

**Published:** 2024-01-12

**Authors:** Tamilarasan Muniyapillai, Karthikeyan Kulothungan, A Aswin, Abinaya R, Guruvenkat G, Hafeeza Kulthum Z, Hajira Beevi M, Harigovindh B

**Affiliations:** 1 Community Medicine, Dhanalakshmi Srinivasan Medical College and Hospital, Perambalur, IND; 2 Community Medicine, Panimalar Medical College Hospital and Research Institute, Chennai, IND

**Keywords:** dietary control, glucose management, self-care, type 2 diabetes mellitus, diabetes self-management questionnaire

## Abstract

Background

The complications of type 2 diabetes mellitus (T2DM) continue to cause significant morbidity and mortality, resulting in a substantial economic burden on both individual patients and society. The adoption of self-care practices leads to enhanced glycemic control, decreased complications, and an elevated quality of life. This study aimed to examine self-care activities and their association with glycemic control among individuals with diabetes.

Materials and methods

A cross-sectional study was conducted, involving 150 previously diagnosed T2DM patients who visited the tertiary care hospital in Perambalur, Tamilnadu, India, from March 2023 to May 2023. The collection of data involved conducting a semi-structured interview using the diabetes self-management questionnaire (DSMQ) over an eight-week period. Following the input of the data into MS Excel (Microsoft® Corp., Redmond, WA), SPSS Statistics version 26.0 (IBM Corp. Released 2019. IBM SPSS Statistics for Windows, Version 26.0. Armonk, NY: IBM Corp) was used for data analysis.

Results

The mean age of the patients was 58.35±11.97 years, and 54.7% (n=82) of them were male. Nearly 65% of diabetic patients (n=98) were on orally administered antihyperglycemic agents. Fifty-nine percent (n=89) of the patients were observed to possess self-care behaviors that met the criteria for adequacy, as the DSMQ scores were dichotomized into "adequate" (≥6) and "inadequate" (<6) categories. We observed that 65% (n=98) of the patients had uncontrolled T2DM, characterized by an HbA1C level above 7.5%. Out of the four subscales of self-care behaviors assessed in this study, "glucose management" scored the highest (5.27±1.30), followed by "dietary control" (5.09±1.53), "healthcare use" (4.86±1.50), and "physical activity" (3.27±1.42). The proportion of diabetic patients who had adequate self-management (55%, n=49) had better glycemic control compared to diabetic patients who had inadequate self-management (4.91%, n=3), and this difference in proportion was statistically significant by the chi-square test (p-value 0.001). Similarly, a statistically significant association was noted between glycemic control and the subscales of DSMQ, namely glucose management, dietary control, physical activity, and healthcare utilization.

Conclusion

The findings in this study indicate that a noticeable proportion of T2DM patients practice inadequate self-care behaviors. According to the DSMQ, diabetic patients with adequate self-management had better glycemic control than diabetic patients with inadequate self-management. According to this research, patients with good glycemic control also tend to exercise better self-care management and show a greater concern for their illness.

## Introduction

Diabetes mellitus is a chronic and progressive metabolic disorder characterized by elevated blood glucose levels (hyperglycemia) resulting from either an absolute or relative deficiency of the insulin hormone [1]. According to the International Diabetes Federation (IDF), 537 million individuals aged 20-79 years were affected by diabetes in 2021. By 2030, experts expect this figure to reach 643 million and project it to further rise to 783 million by 2045. Diabetes is associated with complications such as cardiovascular diseases, nephropathy, retinopathy, and neuropathy, which can lead to chronic morbidities and mortality [2]. Diabetes accounts for roughly 6.7 million deaths on a global scale [3]. The occurrence of diabetes-related deaths is highest in low- and middle-income countries, accounting for almost 80% of cases [2].

The prevalence of diabetes in India has seen a rise from 7.1% in 2009 to 8.9% in 2019 [4]. In the year 2019, the prevalence of type 2 diabetes mellitus (T2DM) in India was approximately 77 million among adults, with nearly 25 million individuals classified as prediabetic [5]. India holds the second position globally for the highest number of T2DM patients, following China, reflecting the massive burden of T2DM in the country [6]. The number of undiagnosed individuals with diabetes in India is approximately 43.9 million, constituting nearly 57% of the adult population [4]. The lack of awareness of diabetic status among over half of the population adds to the complexity of preventing severe complications for both healthcare workers and patients [5].

There has been a significant increase in global health expenditure attributed to diabetes, with the amount growing from USD 232 billion in 2007 to USD 966 billion in 2021 for adults aged 20-79 years [3]. The IDF projects that the total expenditure on healthcare related to diabetes will amount to USD 1.03 trillion by 2030 and USD 1.05 trillion by 2045 [3]. The projected increase in direct costs pertaining to diabetes presents a worldwide public health emergency, posing a threat to the economies of all nations, with a particular emphasis on developing countries and low- to middle-income nations.

Self-care is a term used to describe the intentional endeavors of individuals, families, and communities to enhance health, avert illness, and regain wellness. These activities are based on knowledge and skills gained from both professional and non-professional experiences. Individuals carry out these tasks for their own benefit, either individually or through participatory collaboration with professionals [7].

The presence of diabetes in adults leads to a two- to three-fold increase in the risk of heart attack and stroke. The presence of neuropathy in the feet, accompanied by impaired blood circulation, heightens the probability of developing foot ulcers and subsequent infection, resulting in the need for limb amputation. Long-term damage to the retinal blood vessels gives rise to diabetic retinopathy, a prominent cause of visual impairment and blindness. Diabetes ranks among the primary factors contributing to kidney failure [5].

The nature of diabetes as a non-communicable and chronic disease, with life-threatening consequences and various modifiable and non-modifiable risk factors, causes significant daily self-management by patients besides medical treatment [8-11]. The global recognition of the importance of self-management in the care of chronic diseases is widespread among healthcare professionals [12]. Diabetes self-management involves facilitating the knowledge, skills, and abilities necessary for a patient to manage their condition effectively.

The practice of seven essential self-care activities in individuals with diabetes is associated with better glycemic control, fewer complications, and an improved quality of life [13-15]. The factors include healthy eating, physical activity, blood sugar monitoring, medication compliance, problem-solving abilities, coping skills, and risk-reduction behaviors [2,15]. Self-care encompasses not only the performance of these activities but also the interrelationships that exist among them [16]. Because of the intricate nature of the issue, an exhaustive, diverse, and integrated strategy is crucial in encouraging diabetic patients to practice self-care and prevent long-term effects [2]. Based on the current literature, a cross-sectional study was conducted to examine self-care activities and their association with glycemic control among individuals diagnosed with diabetes.

## Materials and methods

Study design, period, and participants

A cross-sectional study was conducted in a tertiary care hospital in Perambalur, Tamilnadu, South India. We collected data from the patients diagnosed with T2DM who were admitted to the general medicine ward during the study period of six months (March 2023-August 2023).

Inclusion and exclusion criteria

Patients with T2DM who had been diagnosed with the disease for at least a year and who gave their consent to take part were included in this study. This study excluded patients with type 1 diabetes mellitus, pregnant women (including those with gestational diabetes), and diabetic patients who were seriously ill, unable to walk, had foot disabilities, were disoriented, or were unconscious.

Sample size and sampling method

In a cross-sectional study conducted by Ang et al. at a public tertiary care center in Northern Malaysia, they found that 15.5% of diabetic patients displayed inadequate self-care behaviors over a nine-month period [17]. By utilizing the formula N = 3.84 * p * q / d2, where p denotes prevalence, q denotes the complement of p, and d denotes precision (with a 6% absolute error), we calculated the sample size according to the above prevalence rate. It was determined that the study required at least 137 samples. We included all diabetic patients who were admitted to the hospital in consecutive order.

Ethical approval and informed consent

We got ethics approval from the Institutional Ethics Committee of Dhanalakshmi Srinivasan Medical College and Hospital (approval number: IECHS/IRCHS/No. 393) before the commencement of the study. A detailed explanation was provided to each participant, and their written consent was obtained prior to data collection. We ensured that we fully informed the participants of the study's objectives, potential risks, and benefits by obtaining their informed consent prior to conducting the study.

Data collection tools

A semi-structured interview schedule administered by an interviewer was used to collect data on background characteristics, including age, marital status, working status, education, smoking, and alcohol. The patients also provided information on the duration of diabetes, treatment of diabetes mellitus, and family history.

Self-care behaviors were evaluated using the validated and extensively employed self-reported scale, known as the diabetes self-management questionnaire (DSMQ), for the quantification of diabetes self-management. It served as a statistical predictor for monitoring glycemic control in T2DM over the preceding eight-week period [18]. This scale measured self-care behaviors through the use of specific questions related to adherence to medication, regularity of health check-ups, dietary habits, physical activity, home blood sugar testing, foot care, smoking, and alcohol consumption [19]. The DSMQ inventory comprises sixteen self-care items that are divided into four subscales: glucose management (items 1, 4, 6, 10, 12), dietary control (items 2, 5, 9, 13), physical activity (items 8, 11, 15), and healthcare use (items 3, 7, 14). Last, item 16 represents overall self-care. In our rating process, we use a four-item Likert scale, with zero denoting "does not apply to me" and three denoting "applies to me very much." To calculate the scores, the nine negatively phrased items are reversed. A conversion was made to a scale of 0 to 10 for all scores. The higher the score on the scale, the more effective the self-care is [20]. Following the recommendation of Schmitt et al. on the cut-off value, the participants' DMSQ scores were dichotomized into "adequate" (≥6) and "inadequate" (<6) self-care behaviors [20].

A clinical examination was conducted to measure height and weight to calculate the body mass index. The BMI was computed using the formula: BMI = weight (kg) / height in m2. Subsequently, the BMI was classified according to Asian standards, wherein values below 18.5 were categorized as underweight, 18.5-22.9 as normal weight, 23-27.5 as overweight, and >27.5 as obese [21]. To measure the waist circumference, the top of the iliac crest is considered, while we measured the hip circumference at the widest point of the buttocks [22].

We performed the estimation of random blood sugar (RBS) using a standardized glucometer. The most recent measurements of fasting blood sugar (FBS) and post-prandial blood sugar (PPBS), as well as glycosylated hemoglobin (HbA1c), were collected from previous medical reports. In diabetes management, a RBS level above 200, an FBS level above 126, and a PPBS level above 200 show uncontrolled diabetes mellitus [23]. We classified the participants into two groups as either "controlled" (≤7.5%) or "uncontrolled" (>7.5%) T2DM, using their HbA1C levels [17,24].

Statistical analysis

All data were entered in Excel (Microsoft® Corp., Redmond, WA) and analyzed in SPSS Statistics version 26.0 (IBM Corp. Released 2019. IBM SPSS Statistics for Windows, Version 26.0. Armonk, NY: IBM Corp). Categorical variables are represented by frequency and percentage in demographic data, while continuous variables are expressed as mean and standard deviation or median and interquartile range. The examination of the association between DSMQ self-care activities and HbA1c level was assessed by a chi-square test. A p-value was considered significant if it was less than 0.05.

## Results

Out of 150 participants, 54.7% (n=82) were male, and the mean age of the respondents was 58.35±11.98 years. The mean BMI of the study participants was found to be 24.24±3.01 kg/m^2^. Nearly 94.7% of diabetics were married (n=142), leaving about five unmarried and three divorced. Around 33% (n=50) had a history of alcohol consumption, and 25.3% (n=38) had a history of smoking in the past eight weeks. Around 86 diabetic patients have completed primary education (57.3%). Nearly 34% (n=51) were unemployed. The number of subjects who reported no family history of diabetes amounted to 77, which made up 51.3% of the participants. We have described the basic characteristics of the study participants in Table 1.

**Table 1:** Characteristics of study participants

Socio-demographic data		Frequency (%)
Age (in years)		58.35±11.98 (mean±SD)
Gender	Male	82 (54.7%)
	Female	68 (45.3%)
Marital status	Married	142 (94.7%)
	Unmarried	5 (3.3%)
	Divorced	3 (2%)
Alcohol consumption	Yes	50 (33.3%)
	No	100 (66.7%)
Smoking	Yes	38 (25.3%)
	No	112 (74.7%)
Education	No formal education	16 (10.7%)
	Primary education	86 (57.3%)
	Secondary education	33 (22%)
	Graduate education	15 (10%)
Working status	Employed	99 (66%)
	Unemployed	51 (34%)
Family history of diabetes mellitus	Yes	73 (48.7%)
	No	77 (51.3%)
Body mass index (kg/m^2^)		24.24±3.01 (mean±SD)

Nearly 65.3% of the subjects (n=98) were prescribed oral hypoglycemic agents (OHAs) only; patients on insulin and OHA comprise 24.7% (n=24.7), while only 10% (n=15) were on insulin-only drug therapy. We have described the anti-diabetic therapy of the study participants in Table 2.

**Table 2:** Anti-diabetic therapy of the study participants OHA: oral hypoglycemic agents

Anti-diabetic therapy	N (%)
Only insulin	15 (10%)
Insulin and OHA	37 (24.7%)
OHA only	98 (65.3%)

The mean duration of T2DM among the study samples was 7.84±5.92 years. The mean HbA1c level among the study samples was 7.91±1.03. Table 3 shows the descriptive values of HbA1C, FBS, PPBS, and RBS values, respectively.

**Table 3:** Diabetic profile of the study participants IQR: interquartile range, HbA1c: hemoglobin A1C, FBS: fasting blood sugar, PPBS: post-prandial blood sugar

Diabetic profile	Median (IQR)	Mean±SD
Diabetes duration (in years)	6.00 (4.00–10.00)	7.84±5.92
HbA1c (%)	8.00 (7.00–8.00)	7.91±1.03
FBS	165.00 (154.00–196.25)	176.52±38.06
PPBS	254.00 (224.00–295.00)	265.42±57.24
Random blood sugar	186.50 (144.50–242.00)	199.44±61.20

**Figure 1:**
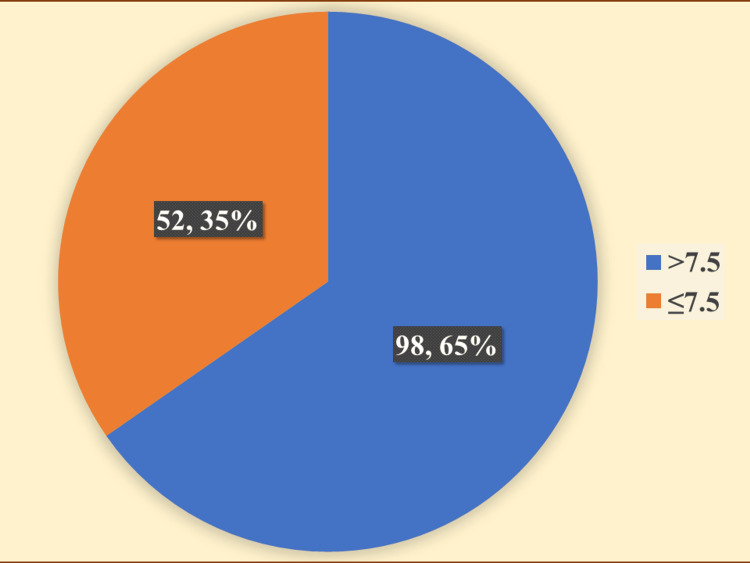
Diabetic status of study participants as categorized by HbA1c value

In the current investigation, we observed that 65% (n=98) of the patients had uncontrolled T2DM, characterized by an HbA1C level above 7.5%. Figure 1 displays the diabetic status of study participants as categorized by HbA1c value.

**Figure 2:**
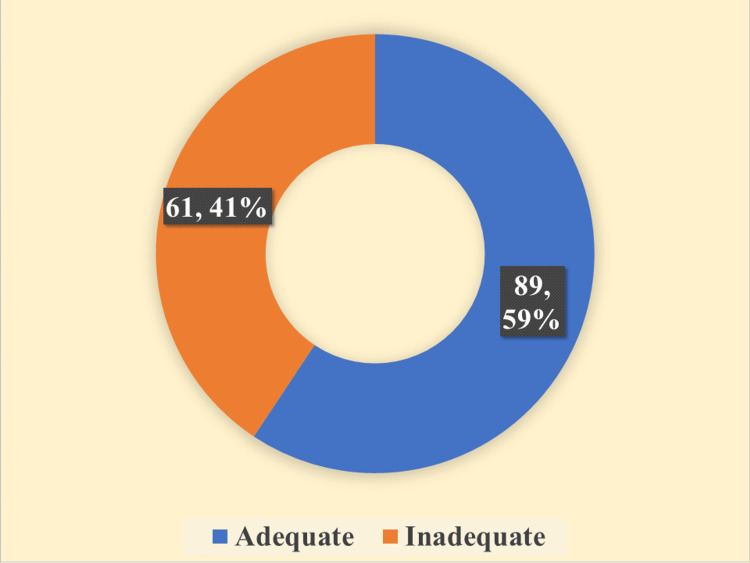
Distribution of self-care behavior exhibited by the diabetic patients

According to the findings of this study, 59.3% (n=89) of the patients were observed to possess self-care behaviors that met the criteria for adequacy, as the DSMQ scores were dichotomized into "adequate" (≥6) and "inadequate" (<6) categories. Figure 2 depicts the distribution of self-care behaviors exhibited by diabetic patients.

Table 4 shows the descriptive statistics of the DSMQ Score and their subscales.

**Table 4:** Descriptive statistics of the DSMQ score IQR: interquartile range, DSMQ: diabetes self-management questionnaire

Variable	Median (IQR)	Mean±SD
Sum value of DSMQ	4.58 (3.95–5.00)	4.58±0.82
Sub-scale of DSMQ		
Glucose management	5.33 (4.50–6.00)	5.27±1.30
Dietary control	5.00 (4.17–6.67)	5.09±1.53
Physical activity	3.33 (2.22–3.33)	3.27±1.42
Healthcare use	4.44 (3.33–5.55)	4.86±1.50

Following the categorization of participants into two groups according to their HbA1c values, the chi-square test was applied to validate the known groups. The patients' glycemic control was categorized based on their HbA1c values into two groups: “glycemic control achieved (≤7.5%) and “glycemic control not achieved (>7.5%)” groups. Table 5 shows the association between the DSMQ score and glycemic control using HbA1c levels. A higher proportion of diabetic patients (58%, n=18) who effectively managed their glucose exhibited improved glycemic control compared to those (28.5%, n=34) with inadequate glucose management. The chi-square test (p-value 0.002) confirmed the statistical significance of this difference. Diabetic patients who had adequate dietary control (50.6%, n=42) exhibited better glycemic control compared to those with inadequate dietary control (14.9%, n=10), and this difference in proportion was found to be statistically significant through a chi-square test (p-value 0.001). A statistically significant difference in proportion was observed between diabetic patients with adequate physical activity (51.2%, n=40) and those with inadequate physical activity (16.6%, n=12) in terms of glycemic control. The chi-square test yielded a p-value of 0.001. There was a statistically significant difference in glycemic control between diabetic patients with adequate healthcare utilization (51.75%, n=4) and those with inadequate healthcare utilization (11.1%, n=7), as determined by the chi-square test (p-value 0.001). The diabetic patients who showed adequate self-management (55%, n=49) exhibited superior glycemic control compared to those with inadequate self-management (4.91%, n=3). This difference in proportion was found to be statistically significant through the chi-square test (p-value 0.001).

**Table 5:** Comparison of the DSMQ self-care activities in patients with HbA1c level DSMQ: diabetes self-management questionnaire

DSMQ		Glycemic control		Chi-square value	p-value
		Achieved (N=52)	Not achieved (N=98)		
Glucose management	Adequate	18 (58%)	13 (42%)	9.445	0.002
	Inadequate	34 (28.5%)	85 (71.5%)		
Dietary control	Adequate	42 (50.6%)	41 (49.4%)	20.834	0.001
	Inadequate	10 (14.9%)	57 (85.1%)		
Physical activity	Adequate	40 (51.2%)	38 (48.7%)	19.807	0.001
	Inadequate	12 (16.6%)	60 (83.3%)		
Healthcare use	Adequate	45 (51.7%)	42 (48.2%)	26.616	0.001
	Inadequate	7 (11.1%)	56 (88.8%)		
Sum scale	Adequate	49 (55%)	40 (44.9%)	40.171	0.001
	Inadequate	3 (4.91%)	58 (95%)		

## Discussion

This cross-sectional study took place at a tertiary care medical college and hospital in Tamilnadu, South India. It aimed to investigate self-care practices among patients with DSMQ. Diabetes self-care encompasses a wide range of activities, given its multi-dimensional nature. It is of utmost importance to evaluate each part independently rather than consolidating scores across parts. The present study employed a standard DSMQ questionnaire to measure various aspects of diabetic self-care, including dietary control, glucose management, physical activity, and healthcare utilization.

Among the 150 samples analyzed, the gender distribution revealed that 54.7% (n=82) were male and 45.3% (n=68) were female, which is consistent with the findings of a study conducted by Khan et al. in 2021 at the outpatients department in the Rural Health Training Centre in Lucknow, where males made up 53% and females made up 47% [8]. In a study conducted by Kakade et al. in 2016, they examined diabetic patients attending a tertiary care hospital in Navi Mumbai. The study revealed that the population comprised approximately 56.7% males and 43.3% females [25]. According to a study performed by Padma et al. in India, they included 117 diabetic patients attending tertiary hospitals, with 63 (53.85%) being male and 54 (46.15%) female [26].

The mean age in our study is 58.35 years, whereas the study conducted by Khan et al. in Saudi Arabia in 2021 reported an average age of 53.37 years [8]. As per a study conducted by Shah et al. in India, they observed that the mean age of diabetic patients attending tertiary hospitals was 55.82±10.2 years [27]. Healthcare providers may perceive the older adult population as vulnerable because of the presence of several ailments and potential limitations in self-care activities, which could explain the findings.

A considerable proportion of the subjects (65.3%, n=98) in the present study were prescribed OHA's only; patients on insulin and OHA comprise 24.7% (n=37), while a smaller percentage (10%, n=15) were on insulin-only drug therapy. In a study carried out at Awan Medical Complex, a tertiary hospital in Lahore, Pakistan, Bukhsh et al. identified that 45% of patients used OHAs alone or in combination with insulin (44%), whereas 11% only used insulin [28].

In the present study, we observed that 65% (n=98) of the patients had uncontrolled T2DM, characterized by an HbA1C level above 7.5%. A similar finding was noted in a 2016 study conducted at Awan Medical Complex, a tertiary hospital in Lahore, Pakistan. Bukhsh et al. noticed that 62% of the patients had HbA1c values higher than 7.5% [28]. In a 2018 study at a public tertiary care hospital in northern Malaysia, Ang et al. observed that 44.7% of patients had uncontrolled T2DM, characterized by an HbA1C level above 7.5% [17]. Therefore, further investigation is needed to understand the factors contributing to uncontrolled T2DM in India.

In the present study, among the four subscales of self-care behaviors evaluated, “glucose management” had the highest score (5.27±1.30), followed by “dietary control” (5.09±1.53), “healthcare use” (4.86±1.50), and “physical activity” (3.27±1.42). A similar finding was noted in a 2016 study conducted at Awan Medical Complex, a tertiary hospital in Lahore, Pakistan. Bukhsh et al. noticed that glucose management had the highest score (5.3±2.9) than other domains [28]. In a study published in 2018 at a public tertiary care hospital in northern Malaysia, Ang et al. found that “healthcare use” had the highest score (8.36±1.99), followed by “glucose management” (7.61±2.56), “dietary control” (7.49±1.89), and “physical activity” [17]. We observed a similar pattern in the 2013 study conducted at a German tertiary referral center by Schmitt et al. in which healthcare use had the highest score than other domains [20]. The increase in self-care activity scores could be because of the difficult COVID-19-related circumstances, which made people more attentive to their lifestyle and well-being.

In the present study, diabetic patients with adequate "glucose management," "dietary control," "physical activity," and "healthcare use" reported significantly better glycemic control than inadequate groups, as measured by the DSMQ. Correspondingly, the proportion of diabetic patients who had adequate self-management had better glycemic control compared to diabetic patients who had inadequate self-management. We observed similar findings in a 2013 study conducted by Schmitt et al. at a German tertiary referral center. When comparing patient groups based on glycemic control, there are three categories: "good" (HbA1c≤7.5%), "medium" (HbA1c 7.6-8.9%), and "poor" glycemic control (HbA1c≥9.0%). They revealed significant differences regarding both the DSMQ sum scores and the subscale scores [20].

The contrast result was seen in a cross-sectional study by Mehraver et al. in 2014 among 562 Iranian T2DM patients, and they did not find any relationship between HbA1c levels and diabetes mellitus self-management [29]. The results indicate that patients who engage in good self-care and show concern for their disease achieve better glycemic control and have a lower likelihood of experiencing complications related to diabetes.

Limitations

Considering the constraints on resources, we used convenient sampling to conduct this study among diabetic patients who visited the tertiary care hospital in Tamilnadu, South India. The findings could not be generalized to other regions of the country because of the study's exclusive focus on participants from a specific geographic area. By conducting a multi-centric study using a probability sampling technique throughout Tamilnadu, more favorable results could be obtained. It should be recognized that the assessment, even though conducted using the DSMQ scale, remains subjective in nature and was solely assessed by individuals who visit the hospital, potentially predisposing them to possess a positive attitude toward the management of the disease. Therefore, the evaluation of self-care behaviors was solely reliant on self-reported data provided by the participants, which could be influenced by bias resulting from social desirability or selective recall bias. The association identified in this study may not be causal, given the cross-sectional nature of the study.

## Conclusions

The findings in this study imply that a noticeable proportion of patients with T2DM had inadequate self-care behaviors. We should make continuous efforts to educate individuals with diabetes about these inadequate behaviors, as they play a vital role in preventing complications and improving quality of life. In this study, diabetic patients with adequate "glucose management," "dietary control," "physical activity," and "healthcare use" reported significantly better glycemic control than inadequate groups, as measured by the DSMQ. Correspondingly, the proportion of diabetic patients who had adequate self-management had better glycemic control compared to diabetic patients who had inadequate self-management. The critical impact of managing blood sugar levels, nutrition, exercise, and medical utilization collectively is highlighted in this research as a key factor in maintaining glycemic control.
